# A Wireless Portable High Temperature Data Monitor for Tunnel Ovens

**DOI:** 10.3390/s140814712

**Published:** 2014-08-12

**Authors:** Ricardo Mayo Bayón, Víctor M. González Suárez, Felipe Mateos Martín, Juan M. Lopera Ronda, Juan C. Álvarez Antón

**Affiliations:** Department of Electrical Engineering, Viesques Campus, University of Oviedo, Gijón 33204, Spain; E-Mails: victor@isa.uniovi.es (V.M.G.S.); felipe@isa.uniovi.es (F.M.M.); lopera@uniovi.es (J.M.L.R.); anton@uniovi.es (J.C.Á.A.)

**Keywords:** food processing industry, tunnel ovens, biscuit, datalogger, zigbee

## Abstract

Tunnel ovens are widely used in the food industry to produce biscuits and pastries. In order to obtain a high quality product, it is very important to control the heat transferred to each piece of dough during baking. This paper proposes an innovative, non-distorting, low cost wireless temperature measurement system, called “eBiscuit”, which, due to its size, format and location in the metal rack conveyor belt in the oven, is able to measure the temperature a real biscuit experience while baking. The temperature conditions inside the oven are over 200 °C for several minutes, which could damage the “eBiscuit” electronics. This paper compares several thermal insulating materials that can be used in order to avoid exceeding the maximum operational conditions (80 °C) in the interior of the “eBiscuit. The data registered is then transmitted to a base station where information can be processed to obtain an oven model. The experimental results with real tunnel ovens confirm its good performance, which allows detecting production anomalies early on.

## 1. Introduction

**Figure 1.**
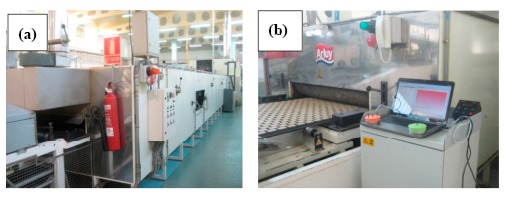
(**a**) Exterior view of a tunnel oven; (**b**) Entrance to a tunnel oven.

In many industrial sectors, it is necessary to maintain critical variables with a high degree of accuracy and uniformity. The food industry dedicated to the production of biscuits and pastries is an example. In this sector, tunnel ovens are used for the baking process. Tunnel ovens are designed for very large, fully automated, continuous processes and they can reach a length of up to 150 m, with temperatures ranging between 200 °C and 250 °C. A tunnel oven can be seen in Figure 1a,b.

In normal operation the product is placed on a conveyor that moves it from assembly through baking to cooling and packaging. Tunnel ovens can use direct-fired convection heating, indirect-fired heating and in some cases, radiant heating if a browning or crisping enhancement section is needed [1].

In direct-fired convection ovens, the heat is transferred to the dough using hot air. The air is heated directly (without a heat exchanger) between 220 °C and 250 °C and it is directed into the oven compartment where it spreads over the top and bottom of the product.

In indirect convection ovens, the heat is transferred to the dough using clean (uncontaminated) hot air. This air reaches a high temperature using a group of burners and a heat exchanger to avoid any contamination of the air that comes into contact with the cooked product in the oven. The air moves across the top and bottom of the product and can normally reach a temperature of around 200 °C in the oven compartment.

Indirect fired heating ovens have an oven compartment comprised of tubes placed above and below the transporter. These tubes transfer the heat to the dough. A group of burners heats the air, which is forced through the tubes by a mechanical fan.

Lastly, direct gas ovens use a high number of burners with the flame located directly in the oven compartment. The heat is transferred to the product mainly by a radiation effect, and given that there is no metal structure to separate the heat source from the product, these ovens can be considered highly efficient in terms of thermal transfer. This type of oven is especially suited to cooking products that need a short cooking time and high initial temperature. There are also ovens with hybrid configurations, for example mixed ovens that combine radiation and convection to deliver different effects on the biscuit being produced. Optimal and strict control of the temperature profile of the oven will mean a significant reduction in energy consumption and will enable a product of the desired quality to be obtained.

The product, in the form of a biscuit, rests on a metal rack conveyor belt. This configuration allows the heat to circulate around the product during its journey through the oven, a process that may take 2 to 8 min. To achieve a satisfactory final product, it is essential to control certain critical parameters such as temperature, humidity, air speed and heat transfer during the baking. These variables, especially oven temperature, are critical for the quality of the final product [2,3].

In general, any temperature variability between relatively close points within the oven jeopardizes product uniformity due to the different way heat is applied. Temperature distributions can lead to problems with the final product due to insufficient system adjustment [4].

Thus, either insufficient or excessive temperature during cooking can lead to fermentation and humidity level differences, which can produce changes in colour, texture and taste [5,6]. Those changes can be responsible for considerable losses during production [7].

Moreover, there can be other problems such as variations involved during the first and last bake of the day where the temperature adjustment has not reached optimal levels. This can generate problems in the process that may significantly reduce production until its cause is located. As a consequence, permanent and sustained supervision by experienced factory workers is required [8].

Measurement of the aforementioned physical variables is required in order to automate the process. However, the size and complexity of these production systems hinders the presence of on-line measurement tools in each one of the significant points of the process and constitutes one of the biggest problems in detecting and correcting problematic production conditions [9].

The normal method of temperature measurement that is set up permanently in these ovens is designed to guarantee the temperature of the fluid heat being transmitted, but not the temperature of the final product, or rather, the temperature of the biscuit during its journey through the oven.

The most common temperature sensor is a thermocouple that simply measures the temperature of the air inside the oven. However, there can be discrepancies of up to 50 °C away from the real product temperature, depending on variables such as the airflow and other oven attachments. Besides, these thermocouples react slowly to changes in heat.

Currently, there is a tendency to fit out new ovens with pyrometers, which in contrast to thermocouples allow direct temperature measurement of the product's surface, reflecting any changes in temperature almost immediately. This allows for an exact representation of the heating taking place inside the oven, and contributes to optimal control of the process.

In addition, pyrometers have the advantage of not being subject to thermal drifts or wastage. A pyrometer immediately detects the lowest temperature of a product that enters the oven and allows the cooking process to be adjusted accordingly.

The biggest drawback of pyrometers is their high cost and the fact that it is difficult to find space for them in the limited space inside an oven. Moreover, they are only able to take the temperature in the immediate space around where they are installed.

Therefore, there need to be many of them installed throughout the oven to be able to cover a wider space. This drawback means that pyrometers are not suited to long ovens where the biscuit dough is in constant movement.

Therefore, given the drawbacks of fixed means of temperature measurement (thermocouples and pyrometers) to determine the oven profile, biscuit manufacturers often use external, non-fixed means such as data measurement and storage equipment (dataloggers) as their main way of knowing about and controlling the fundamental variables involved in production.

There are a number of wireless devices in the field of temperature measurement and storage (dataloggers), but their use is limited to certain types of installations that bear no resemblance to tunnel ovens because they have some of the following drawbacks: they are not very robust, they have limited measuring capabilities, low autonomy, an inability to adapt to other sensors, or a lack of integrated software for data representation and analysis [10,11].

Moreover, commercial solutions for measuring temperature profiles in tunnel ovens used for biscuit production tend to be managed by external companies and can be very costly if used on a continuous basis [12]. Thus, the most advanced measurement devices are not usually controlled by staff at the factory, but rather by specialised companies offering data capture services, which then analyse the data collected.

Besides, the size and operating mode of these devices makes their usage very complicated when they have to fit in with the factory's usual operating routine, as well as interfering with or altering the variables of the process because of their size or what they are made from. This means that the data produced is not very reliable at times.

To sum up, the use of thermocouples is not very effective and does not reflect the exact temperature of the dough for the reasons heretofore explained. Installing pyrometers is expensive and only provides temperature measurements in one place, not throughout the whole oven, as well as requiring space that is not typically available in the oven.

On the other hand, using external dataloggers is an expensive solution and depends on external personnel. Existing tools on the market do not fit correctly inside the ovens, distorting readings taken from the dough. Another drawback is the difficulty of fitting these devices in a moving oven with the dough on the cooking platform.

Due to all these factors, this paper proposes a solution using the “eBiscuit” that will solve the problems mentioned above [13,14]. The “eBiscuit” is a wireless datalogger, similar to a biscuit in size and format, which rests in a metal rack in a conveyor belt in the oven. This system makes it possible to register the temperature data the real biscuits are experiencing along the conveyor belt. Due to the high temperature the “eBiscuit” is subject to, the thermal insulating materials used for the structure must ensure a maximum inner temperature of 80 °C, which is critical for the proper functioning of the electronic system. This paper analyses several thermal insulating materials which not only guarantee the proper functioning but also comply with the size and format requirements of the “eBiscuit”.

## 2. System Architecture

**Figure 2.**
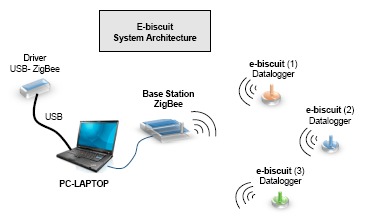
General system architecture.

The architecture of the system can be seen in Figure 2. On the right, there are examples of the “eBiscuit” dataloggers, which have wireless transmission capabilities. These “eBiscuit” record data about temperature as they travel through the oven. On the left, there is the base station that sends and receives information to and from the datalogger, as well as communicating with the PC for further analysis of the collected data [15,16].

### 2.1. System Specifications

The physical constrains of tunnel ovens impose very demanding system specifications on the “eBiscuit” datalogger, such as the maximum height including external sensors, wide exterior temperature range, high operating temperature, and long residence time inside the oven. All these characteristics are shown in Table 1.

From the previous specifications it should be noted: (a) the high working temperature to which the equipment will be exposed for a period of 10 min (250 °C), (b) the volume of data that the device must store.

### 2.2. Operation Mode

The operating mode in place together with the data measurement and acquisition systems is as follows: first, a computer is used to plan the group of tests you would like to be carried out in the oven (test ID, oven data, dataloggers to be used, test duration, location on the oven conveyor belt, *etc.*). Once the tests have been configured, they are downloaded to the base station. Next, the base station wirelessly downloads the desired test to each “eBiscuit” sensor. In this way, data acquisition can be carried out using the established configuration without the need for specialised personnel. Finally, once the tests have finished, the captured data is transmitted to the base station by each datalogger for further analysis [17].

## 3. System Design

### 3.1. Thermal Design

The thermal design is based on the temperature specifications laid out in Table 1. The system has to cope with a working temperature of 250 °C for 10 min intervals, which is the maximum temperature and time interval for a biscuit to be baked correctly. The total dimensions of the casing are limited to a diameter of 90 mm and a width of 30 mm, enabling them to fit inside tunnel ovens (which have limited headspace), and ensuring they do not affect their surroundings.

**Figure 3.**
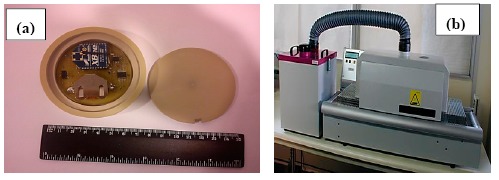
(**a**) PEEK casing “eBiscuit”; (**b**) Test oven.

**Figure 4.**
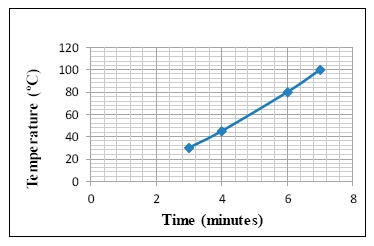
Temperature evolution of an “eBiscuit” with PEEK casing.

The first prototype casing was made of PEEK, see Figure 3, without using any other type of thermal insulation. Different tests were carried out using an FC200 test oven (type F31130) to determine heat transfer across the casing by measuring the internal temperature of the prototype. Figure 4 shows the result of a characteristic test measuring the temperature inside the prototype performed at a test oven at 200 °C.

**Figure 5.**
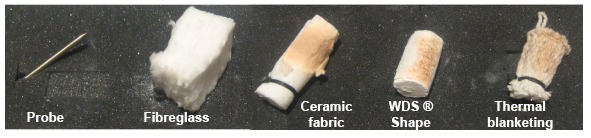
Insulating materials tested: ceramic fabric, WDS^®^ Shape, thermal blanketing, and fibreglass.

As can be seen in Figure 4, the temperature inside the “eBiscuit” reached 80 °C in 3 min. This is not acceptable for a datalogger to be used inside a tunnel oven, as its electronic system could get damaged if this temperature is reached at any moment during the baking process, which is estimated to last an average time of 10 min. A second prototype was designed and built using a PEEK casing with the interior filled with an insulating material. Tests were carried out using various types of insulation to ascertain the best-suited material, see Figure 5:

(a) Fibreglass.
(b) Ceramic fabric.
(c) WDS^®^ Shape.
(d) Thermal blanketing.

**Figure 6.**
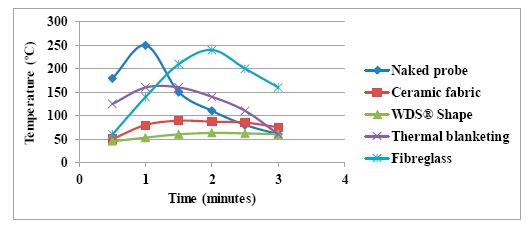
Test results.

In order to check the insulating capacity of the different materials, a Resistance Temperature Detector (RTD) probe was placed inside an 8 mm thick cylinder made from the different insulating materials. The results obtained after applying hot air with a heat gun at 250 °C from a distance of 8 cm for 1 min are shown in Figure 6.

**Figure 7.**
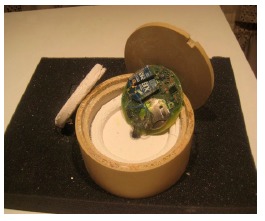
PEEK casing with WDS^®^ Shape insulation prototype.

**Figure 8.**
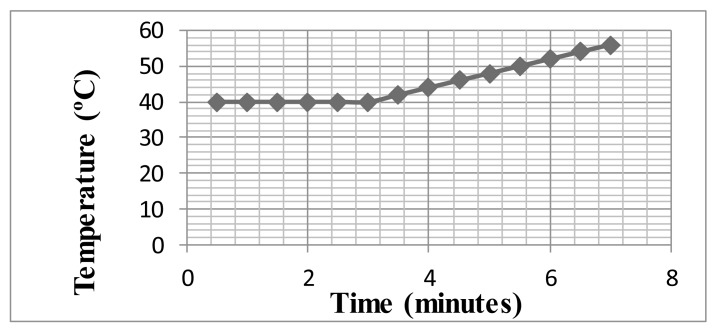
Temperature evolution of “eBiscuit” with Peek casing and WDS^®^ Shape (test at 200 °C).

From the results obtained, seen above, the microporous WDS^®^ Shape material, based on inorganic silicates, was especially able to withstand high temperatures. It also has an extremely low thermal conductivity coefficient, *i.e.*, it has very good insulation properties. Thus, a test was carried out combining both a PEEK casing and WDS^®^ Shape as inside insulating material (Figure 7), with the results seen in Figure 8. We can see that for a period of 7 min, and at a temperature of 200 °C, the internal temperature only reaches less than 60 °C (test carried out in an FC200 oven).

Comparing the temperature evolution charts of Figures 4 and 8 we can conclude that the main insulating property is provided by the WDS^®^ Shape instead of by the PEEK material. However, the WDS^®^ Shape is an extremely fragile material which makes it unsuitable for casing purposes.

**Figure 9.**
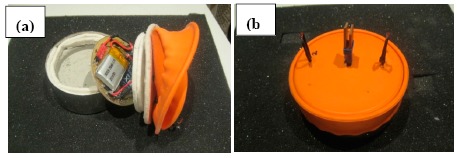
(**a**) “eBiscuit” interior design; (**b**) “eBiscuit” exterior aspect.

So, to try and to reduce the equipment dimensions without affecting its robustness, its insulating capacity and its cost, a new model was designed and built based on a stainless steel casing, and a WDS^®^ Shape insulation on the bottom and sides as can be seen in Figure 9a. The electronic circuit board was covered by a robust but flexible insulating ceramic fabric that was easy to cut and sufficiently solid.

**Figure 10.**
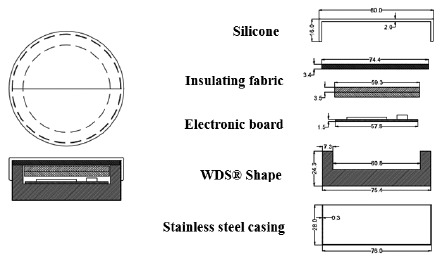
“eBiscuit” design and dimensions.

Finally, the “eBiscuit” was covered with a silicone film (acting as a lid) employed in food industry. Silicone film is generally non-reactive, stable, and resistant to extreme environments and temperatures while still maintaining its properties. The silicone allowed for easy cleaning and location inside the oven, because of its striking colour, as shown in Figure 9b. The “eBiscuit” dimensions are detailed in Figure 10 [18].

### 3.2. Electronic Design

The system is made up of two fundamental modules as shown in Figure 2: the base station and the “eBiscuit” datalogger. These two modules communicate by the Zigbee protocol [19]. The electronic design of these two modules is described in the following sections [20].

#### 3.2.1. Base Station Module

**Figure 11.**
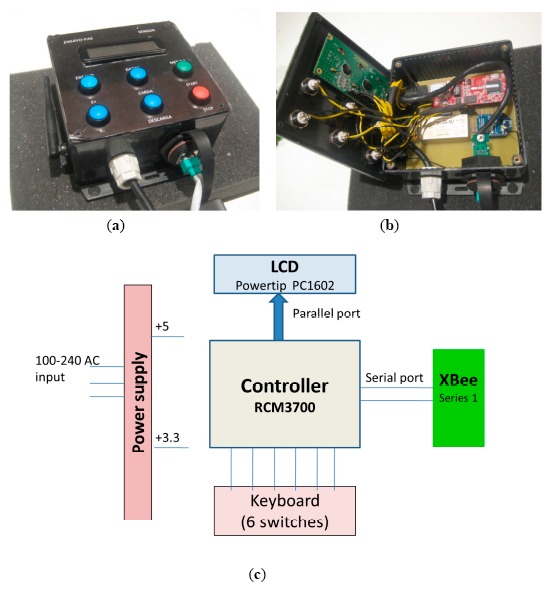
Base station. (**a**) Front view; (**b**) Interior view; (**c**) Electronic schema.

The base station (Figure 11) can be used to wirelessly configure each one of the “eBiscuit” modules. It can also be used to receive and store data from the different “eBiscuit” located inside the oven while it is working. The base station incorporates a microcontroller RCM73000 processing unit, and an integrated Xbee communication device using the Zigbee wireless communication protocol. The microcontroller manages the transmission to the computer through TCP-IP sockets, as well as storing data and configuring tests. The base station also has a user interface made up of a group of buttons and an LCD display so the operator can monitor and control the process.

#### 3.2.2. “eBiscuit” Module

**Figure 12.**
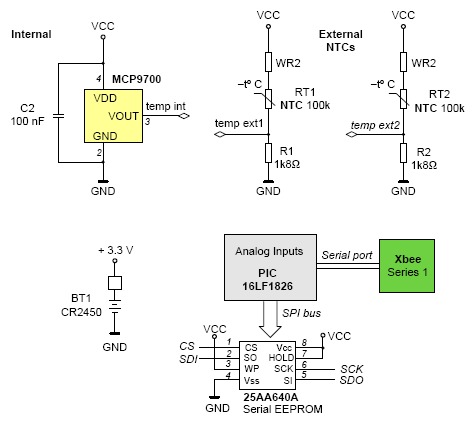
“eBiscuit” electronic circuit.

The heart of the “eBiscuit” contains a PIC 16LF1826 microchip microcontroller (Figure 12). This low power version allows power within the margins of 1.8 V to 3.6 V, with extremely low power usage in standby mode (30 nA). These characteristics make it ideal for use as a portable battery powered device. The device contains a 12 channel A/D converter with 10 bits of resolution.

Up to four sensors can be connected to the converter leaving the rest of the channels free for future extensions. The external temperature sensors are made up of two accurate NTCs. The inside “eBiscuit” temperature is measured by a MCP9700 IC sensor. This linear active thermistor is an analog temperature sensor that converts temperature to analog voltage.

The sensor can measure precisely up to +150 °C and its output is calibrated to a slope of 10 mV/°C. Unlike resistive sensors (such as thermistors), the Linear Active Thermistor IC does not require an additional signal-conditioning circuit. Therefore, the biasing circuit development overhead for thermistor solutions can be avoided by implementing this low-cost device. The voltage output pin (VOUT) can be directly connected to the A/D input of a microcontroller.

The system has also an additional external memory module based on the microchip module 25AA640/25LC640 to store data locally. This memory allows for an extra capacity of 64 K Serial EEPROM. The memory is accessed via a simple Serial Peripheral Interface (SPI)-compatible serial bus.

The system also incorporates a transmission module that guarantees Zigbee communications, both read and write, using the xBee transceiver. This device communicates with the base unit discussed earlier. The microcontroller manages high-level protocol communication, data and digital signal conditioning for temperature sensor. It also deals with calibration functions and alarm management.

**Figure 13.**
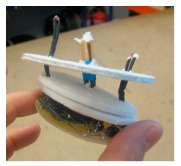
“eBiscuit” printed circuit electronic board.

The “eBiscuit” printed circuit board shape is circular to allow it to be integrated with the most common types of biscuits found in oven lines as can be seen in Figure 13.

The “eBiscuit” is powered by a 3 V lithium button cell battery. However, in the future, other emerging power sources such as the “Harvest Energy” energy generation based on the Peltier effect are expected to be used [21]. This power source may constitute an alternative to conventional batteries.

### 3.3. Software Design

This task relates to the design and development of a software application to capture and use test data with the “eBiscuit” datalogger. It was split up into three main phases: analysis, design, and implementation.

#### 3.3.1. Analysis Phase

During the analysis phase a detailed requirement study, both functional and non-functional, was carried out. Obtaining these requirements is the starting point that allows the different actors that will interact with the system to be identified. It also allows the different subsystems that make up the application to be identified, and allows a use and setting to be defined for each of them. Three actors that will interact with the system have been identified: Client, Sensor and Technician.

▪ The “Client” is the different companies that require test services in their ovens.
▪ The “Sensor” is the external measurement equipment that records data.
▪ The “Technician” is the user application in charge of management, planning and carrying out the tests.

In the same way, there are six main subsystems that will make up the application:

▪ “Companies” subsystem: Brings together all client company data management and maintenance actions.
▪ “Ovens” subsystem: Brings together all actions related to data management and maintenance of analysed ovens.
▪ “Recipes” subsystem: Encompasses all actions related to recipe management and maintenance in each oven as well as of the finished product.
▪ “Sensors” subsystem: Brings together management and maintenance actions of the data collected from measurement sensors.
▪ “Test” subsystem: All necessary actions to manage tests, as well as obtaining and storing data from each test are handled by this subsystem.
▪ “Application configuration” subsystem: This subsystem gathers together all the actions related to staffing the application and its configuration parameters (communications, access to databases *etc.*)

The next step in analysis was to define the static structure of the information system, identifying classes and attributes, and their relationship.

#### 3.3.2. Design Phase

The application design was structured in three layers: presentation, business and data. From a functional point of view, the implementation carried out spans two different blocks:

▪ On one hand, implementing the user interface, the business logic defined during the analysis phase, and the access to the database stored information.
▪ On the other hand, managing the communication with the measurement tools. It was necessary to set up a communication protocol with the measuring tools allowing expert data exchange. This was carried out in the base station to make the system more robust and to make it independent of the PC.

#### 3.3.3. Implementation Phase

**Figure 14.**
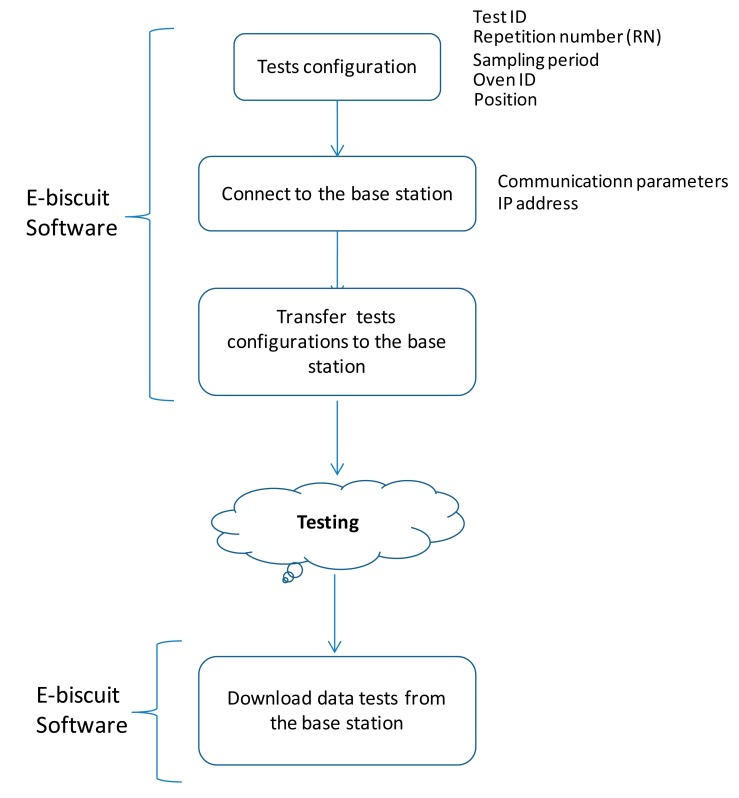
Test sequence diagram.

A software was implemented to cover three type of functionalities identified at the analysis phase: test management, communications between the base station and the “eBiscuit” dataloggers and between the base station and the PC, and finally, report of results. A test can be made up of one or several data captures, which consists of one “eBiscuit” going through a defined tunnel oven. The general sequence of a test can be seen in Figure 14.

**Figure 15.**
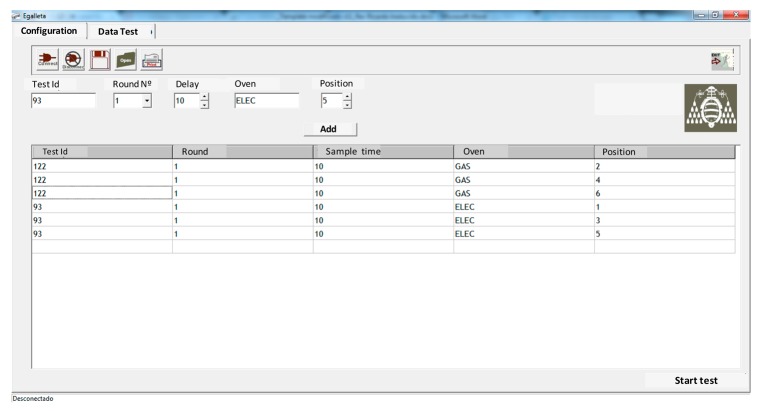
Test configuration

A test is assigned a “Test ID” which uniquely identifies it, and it can be fully defined by five parameters, as seen in Figure 15. The “Position” field indicates the row the “eBiscuit” is located on the conveyor belt. Lastly, the sampling period is shown in tenths of a second.

The test configuration has to be transferred from the PC to the base station (see Figure 14), allowing the non-expert operators to run tests independently from the PC. This provides the system with great flexibility and robustness.

**Figure 16.**
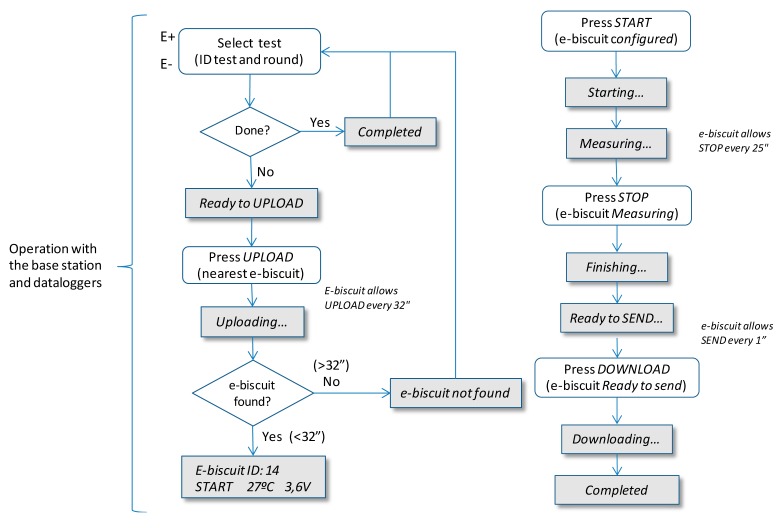
Base station “eBiscuit” communication process.

Then every “eBiscuit” datalogger involved in a given test needs to be configured in order to carry out the test. Figure 16 shows the sequence of the communication between the base station and an “eBiscuit” datalogger.

**Figure 17.**
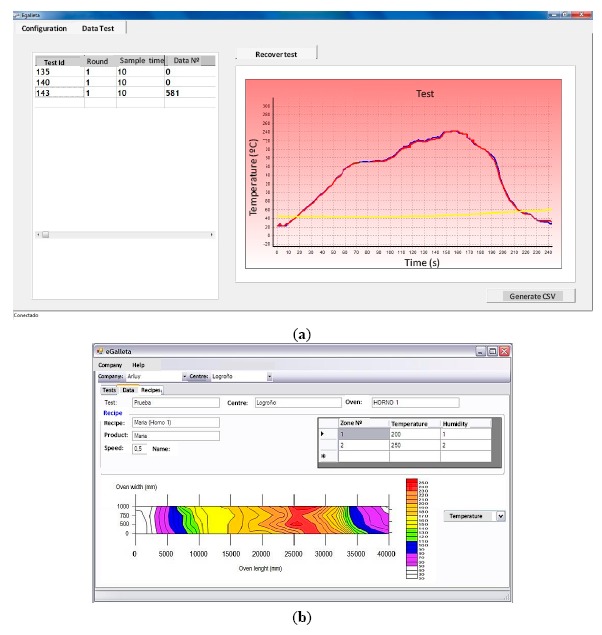
(**a**) Temperature distribution over time; (**b**) Thermal map.

Once a test has been accomplished, the stored data can be downloaded from the “eBiscuit” dataloggers into the base station, and from this into the PC. Based on that data, the application can present the user with results in two different ways: a temperature distribution over time (Figure 17a) and a thermal map (Figure 17b).

Such graphics can also be generated offline from database historic information, enabling for a comparative analysis over time. This provides the expert factory personnel with powerful tools to analyse tunnel oven working conditions, allowing them to detect irregular working conditions, in order to adopt improvement strategies [22].

## 4. Experimental Results

Finally, several tests were carried out and results were collected from two real ovens: a electric tunnel oven (Table 2), and a gas tunnel oven (Table 3).

**Figure 18.**
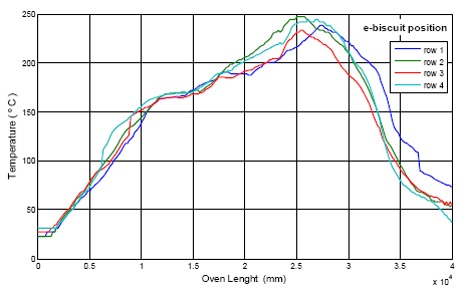
Electric oven temperature distribution.

**Figure 19.**
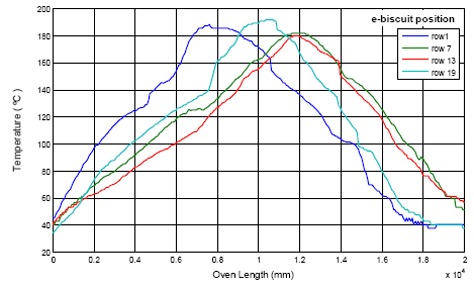
Gas oven temperature distribution.

The tests consisted of four “eBiscuit” data loggers located in four different rows aligned to the same column of the conveyor belt. The test data collected is shown in Figures 18 and 19. They represent the temperature cooking conditions every biscuit is subject to at four different locations inside the oven. It is worth notice the different temperature distribution between both ovens, being more uniform in the electric one.

**Figure 20.**
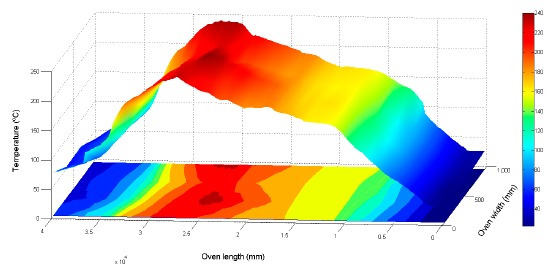
Electric oven 3D temperature distribution.

**Figure 21.**
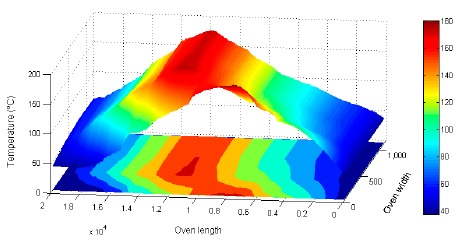
Gas oven 3D temperature distribution.

A 3D presentation of the oven's allocation is shown with the acquired data, in reference to axis X (cooking length of the oven) and axis Y (width of the cooking belt). Results are shown in Figures 20 and 21 [23].

The “eBiscuits” are put on the cooking belt of the electric oven in order to measure its temperature in row 1, 4, 7 and 10, which correspond to following position across the cooking belt: 50 mm, 350 mm, 650 mm and 950 mm respectively.

Furthermore, the “eBiscuits” are put on the cooking belt of the gas oven in order to measure its temperature in row 1, 7, 13 and 10, which correspond to following position across cooking belt: 50 mm, 416 mm, 783 mm and 1150 mm respectively.

## 5. Conclusions

This paper proposes an innovative, portable, non-distorting, low cost, wireless temperature measurement system (“eBiscuit”) for tunnel ovens. Data acquired by the “eBiscuit” is transmitted using Zigbee technology to a base station where information is stored and processed. These are the main features of the “eBiscuit” system:

▪ High temperature. The ovens, where “eBiscuit” dataloggers apply, work at a very high temperature, which make it particularly difficult to select suitable isolating materials. These should protect the electronic hardware of the device from such high temperatures and let it work properly for several minutes.
▪ Non-distorting. A datalogger (“eBiscuit”), which due to its size, format and structure, is able to collect temperatures measurements in different points of the cooking belt of the oven without influencing the environment has been developed.
▪ User friendly. The system includes user-friendly software for test commissioning, historical data management and data analysis. Thanks to the base station, it is possible to work onsite for measurement collection by unspecialized staff.
▪ Low cost. It is a flexible, efficient and low cost solution that can be adapted for use in other high temperature applications.
▪ Decision making. This system helps expert users make early decisions to correct irregular working conditions.

## Figures and Tables

**Table 1.** Design specifications for measuring device (“eBiscuit”).

**DIMENSIONS**	**VALUE**
Electronic dimensions	Diameter: 60 mm; Height: 7 mm
Casing dimensions	Diameter: 90 mm; Height: 30 mm
Maximum height, including sensors	65 mm
Exterior casing material	Compatible with food production environment
Protection class	IP45

**TEMPERATURE SENSOR PARAMETERS**	**VALUE**

No. Exterior temperature sensors	2
Interior temperature sensor	Yes
Exterior temperature range (including preheating)	From 25 to 250 °C
Electronics maximum working temperature	80 °C
Accuracy	2 °C
Resolution	0.1 °C

**OPERATION**	**VALUE**

Maximum attempt-trial time	Up to 10 min
Sampling rate	Up to 10 Hz configurable
Data per attempt-trial	6000 values
Attempt-Trial rate	Minimum: 2

**TRANSMISSION**	**VALUE**

Transmission technology	Zigbee 2.4 GHz, IEEE 802.15.4
Transmission distance	Up to 100 m in line of sight
Compatible receivers	Zigbee-USB PC port

**POWER**	**VALUE**

Voltage	2.8 a 4.2 VDC
Battery life	Up to two years on average with transmission every 10 min
Rechargeable batteries	Optional

**Table 2.** Electric oven parameters.

**PARAMETER**	**VALUE**
Number of sections	4
Temperature Section 1	180 °C
Temperature Section 2	230 °C
Temperature Section 3	240 °C
Temperature Section 4	190 °C
Cooking Time	3.15 min
Oven Length	40 m
Belt Width	1 m
Type of biscuit being produced	Megachoc-500gr
Number of positions/rows	10

**Table 3.** Gas oven parameters.

**PARAMETER**	**VALUE**
Number of sections	2
Temperature Section 1	199 °C
Temperature Section 2	229 °C
Cooking Time	4.5 min
Oven Length	20 m
Belt Width	1.2 m
Type of biscuit being produced	Butter
Number of positions/rows	19
